# Partnering to promote research where it matters

**DOI:** 10.1371/journal.pntd.0005530

**Published:** 2017-04-20

**Authors:** Michael Eisenstein

**Affiliations:** Freelance Science Journalist, Philadelphia, Pennsylvania, United States of America; PLOS, UNITED STATES

Edward Kamau has gone far in his career, earning a PhD in parasitology and a position with the World Health Organization (WHO) Special Programme for Research and Training in Tropical Diseases, or TDR. But he also remembers the challenges he faced as a university student in Kenya in the 1980s. “If you’re bright, you’ll get a government scholarship and your bachelor’s degree—but after that, it’s almost like hitting a brick wall,” he says. While the careers of many of his peers stalled because of limited opportunities, Kamau was talented and fortunate enough to connect with support for postgraduate training in the United States, the United Kingdom, and Kenya.

Today, Kamau (Fig 1) is one of many from his generation who are helping other scientists to excel and building research capacity in low- and middle-income countries (LMICs). The past decades have seen remarkable strides, with traditional “north–south” partnerships, in which Western scientists convey expertise and training to their developing world counterparts, increasingly giving way to regionally based capacity-building efforts and “south–south” collaborations that forge direct alliances between LMIC research communities. Budding researchers also have greater opportunities to read widely, publish, and promote their work, facilitated by a flourishing community of open-access journals that strive to eliminate cost barriers for LMIC scientists. For example, the number of research articles with African authors tripled between 2003 and 2013 [1].

**Fig 1:**
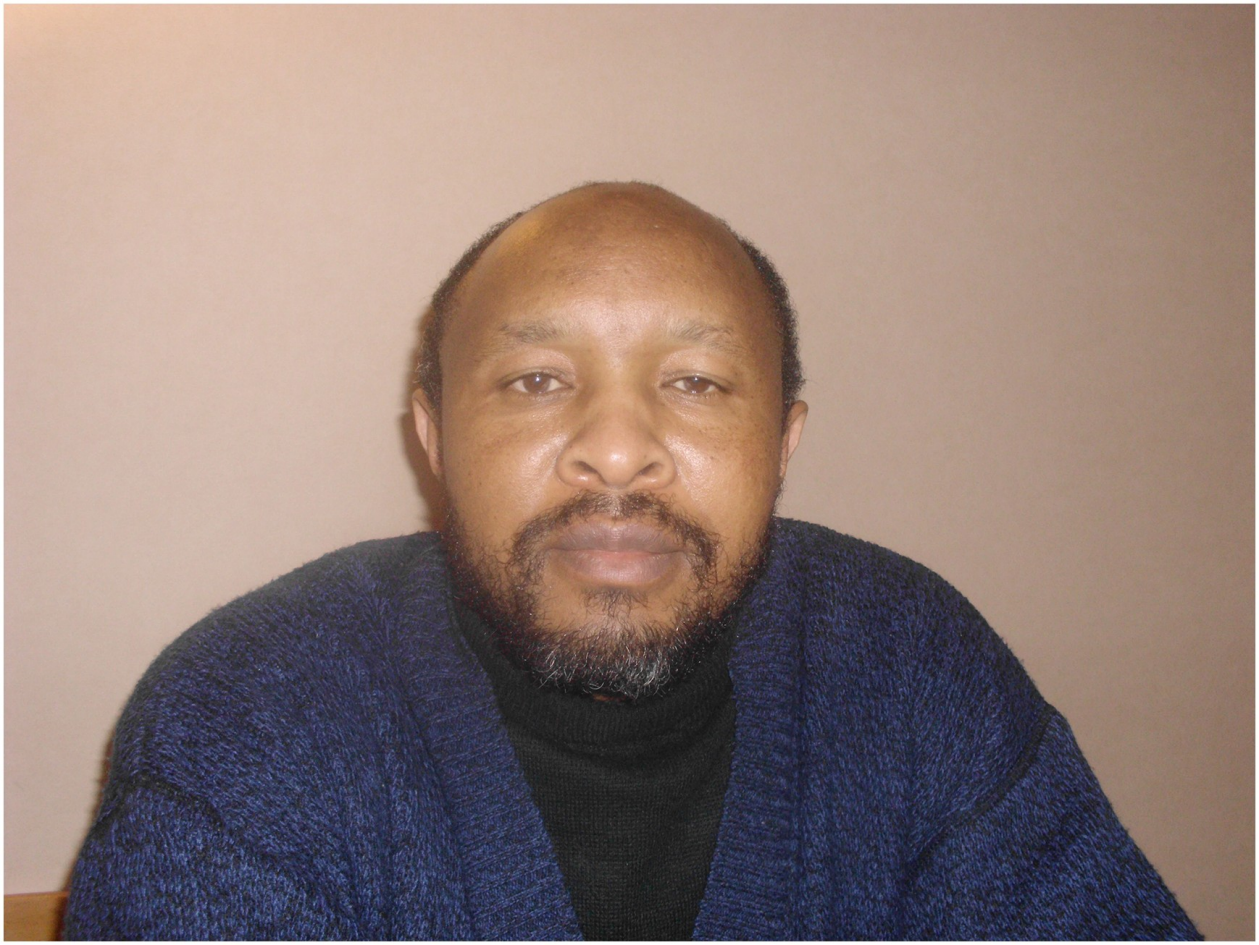
Dr. Edward Kamau is part of the scientific team for the World Health Organization (WHO) Special Programme for Research and Training in Tropical Diseases (TDR) capacity-building program. (Image credit: Dian Paramitasari, Faculty of Medicine, Universitas Gadjah Mada [UGM], Yogyakarta, Indonesia.)

The change is especially striking for neglected tropical diseases (NTDs), which previously languished in the shadow of better-known tropical diseases like malaria. “When I started my work in lymphatic filariasis, the question was, do you really want to have a career?” recalls John Gyapong, Vice Chancellor at the University of Health and Allied Sciences in Ho, Ghana*. Aggressive advocacy has put NTDs on the map, however, and there are now a variety of dedicated research programs and widely-read open-access journals specializing in NTDs—of which *PLOS Neglected Tropical Diseases (PLOS NTDs)* was the first, with a dedicated mission to promote work from LMIC researchers. “We’ve branded these diseases and put them into the context of poverty alleviation,” says David Molyneux of the Liverpool School of Tropical Medicine*. “The future is going to be about building capacity for NTDs and recognizing that we’re talking about a broader problem of sustainable development.”

## Global to local

For decades, international agencies have provided support for students and recent graduates from LMICs to work abroad in the US or Europe. TDR has been at the forefront of such efforts since 1974, and nations like Brazil have grown thriving research ecosystems from its early backing. “In the 1970s and 1980s, TDR made a big impact,” says Carlos Morel, Director of the Center for Technological Development in Health at Brazil’s Oswaldo Cruz Foundation (FIOCRUZ)*. “They were supporting bright students to go abroad and get good training and, when they came back, giving them grants to establish their own labs here.” More recently, TDR has started shifting away from training researchers abroad to forging alliances with well-established academic institutions that can act as regional training hubs. The program currently works with institutions in Bangladesh, Colombia, Ghana, South Africa, Indonesia, Lebanon, and Zambia, and Kamau sees clear advantages to this approach. “The stipends and tuition are lower compared to training in the US or Europe, so maybe you can train five people instead of two,” he says.

**Fig 2:**
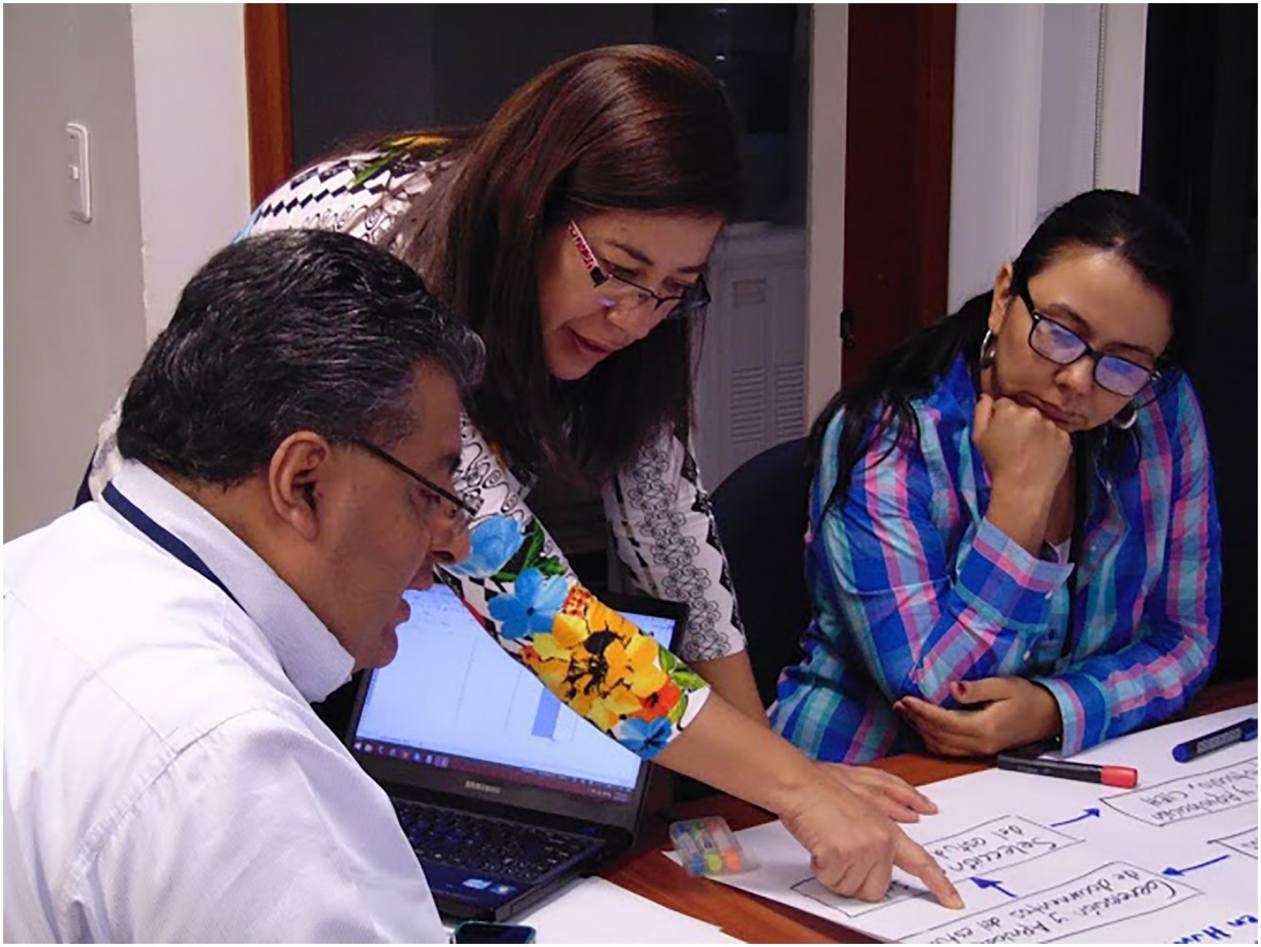
Investigators from diverse backgrounds receive training at a course on Good Clinical Research Practice developed jointly by Centro Internacional de Entrenamiento e Investigaciones Médicas (CIDEIM) and Universidad del Valle in Colombia. (Image credit: Cristian Santacruz).

Nations in Asia, Africa, and Latin America have achieved different levels of autonomy and sophistication in their scientific infrastructure but have mutual enemies in the realm of infectious disease. This has fueled the formation of south–south collaborations that enable newly strengthened research communities to share relevant experience. Brazil’s FIOCRUZ has been fighting NTDs for over a century and is now a research powerhouse with influence throughout the Americas—for example, taking the lead in the recent Zika outbreak. “We are known as a center for training people in science, but also in health management, governance, and things like that,” says Morel. Colombia’s Centro Internacional de Entrenamiento e Investigaciones Médicas (CIDEIM) has also accumulated decades of expertise in capacity building and is now a Regional Training Center funded by the TDR (Fig 2). CIDEIM uses a specialized training program to instruct participants in proper conduct of biomedical research—and gives them the ability to pass that education on. “The modality is ‘training a trainer,’ so others can multiply it in their own setting,” says Scientific Director Nancy Saravia*. “Then they become hubs and spread it.” For example, CIDEIM's program has given rise to parallel programs at the Universidad Nacional Autónoma de Honduras and the Pontificia Universidad Católica del Ecuador.

China is one of the scientific success stories of the 21st century and has achieved major progress in combating NTDs. In 2015, the nation launched the Institution-Based Network for China-Africa Cooperation on Schistosomiasis Elimination to share its expertise in grappling with this parasite. “We ran a training class, and participants from key institutions from nine African countries came to China and learned what we are working on in the laboratory and in the field,” says Xiao-Nong Zhou, Director of the National Institute of Parasitic Diseases at the Chinese Center for Disease Control and Prevention*. China has also helped assemble a large regional network of Asian countries to fight schistosomiasis and liver fluke. “We work together on issues like health education, behavior change, and communication skills,” says Zhou.

Several high-powered African-led research networks have also emerged. The European Foundation Initiative into African Research in Neglected Tropical Diseases recently transitioned its research-funding activities to the Ghana-based African Research Network on NTDs, which is run by African researchers. The European & Developing Countries Clinical Trials Partnership (EDCTP), an alliance to accelerate testing and development of diagnostics, drugs, and vaccines, has also established multiple “regional networks of excellence” to promote collaboration on the African continent. EDCTP has put considerable effort into strengthening self-sufficiency for clinical health research. “We have supported capacity strengthening for ethics and regulation in countries like Rwanda, Mozambique, Togo, Gabon, and Liberia,” says Executive Director Michael Makanga. “Funding from the EDCTP has helped to make their institutional and national committees more functional, and since 2005 we have provided 78 grants in the area of ethics.”

## Share the wealth

**Fig 3:**
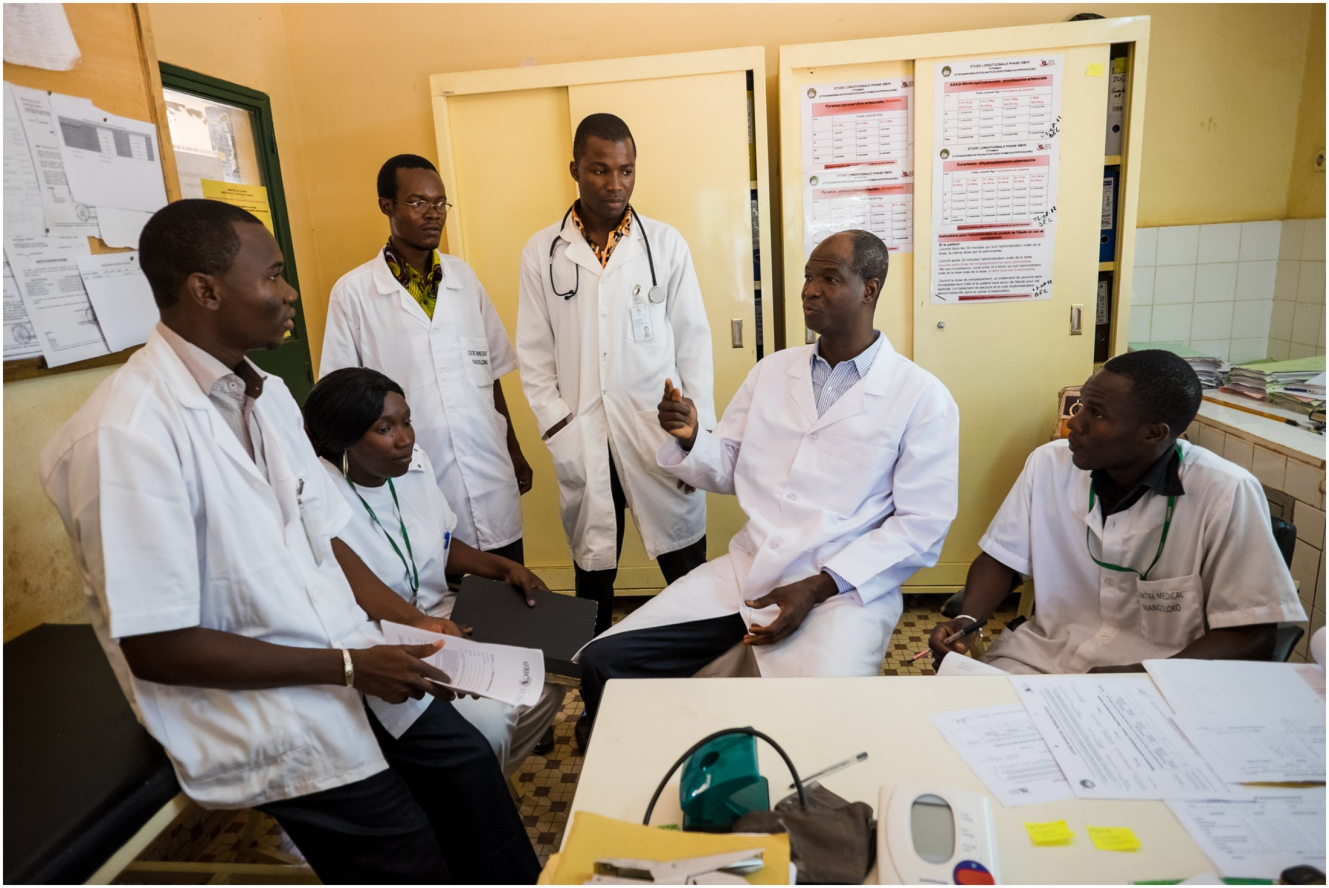
Dr. Sodiomon Sirima's team at the Centre National de Recherche et de Formation sur le Paludisme in Burkina Faso is conducting clinical research into malaria prevention with support from the European & Developing Countries Clinical Trials Partnership (EDCTP). (Image credit: Africa Interactive).

In addition to insights into disease pathology and epidemiology, these capacity-building efforts have driven some decisive victories against the NTDs. “Togo is now getting to elimination of lymphatic filariasis, as is Ghana, and although Kenya’s program faced setbacks, it is now moving forward at a quick pace,” says Mwele Malecela, chief research scientist at Tanzania’s National Institute for Medical Research (NIMR)*. “There has been tremendous progress over the continent.” African researchers are also collaborating with Western partners to build a robust clinical trial infrastructure. EDCTP has played a key role in this area, launching successful African studies of antiretroviral and antimalarial treatment regimens (Fig 3), and under Makanga’s leadership, the organization is also taking aim at NTDs. “Our main priorities will be trials of combination therapies and product-focused implementation research to identify the most effective ways to deliver treatments in particular settings,” he says.

Disseminating research findings has historically been problematic for LMICs, with exasperating barriers limiting access to the literature—both as readers and contributors. “Our university could only afford a very restricted number of titles,” says Nilanthi de Silva, a parasitologist at the University of Kelaniya in Sri Lanka*. “Especially in the medical field, some of the subscription prices are horrendous.” This left many countries walled off from the global research community. On the other side, prohibitive page charges and other fees could pose an insurmountable obstacle for would-be authors without financial support from a Western collaborator.

Open access has proven a transformative force, and many leading NTD journals are not only freely available online but also staffed by editorial board members who come from and actively promote quality research from disease-endemic countries. As Editor-in-Chief of *Infectious Diseases of Poverty*, Zhou sees capacity building as a primary objective. “About 60% of our journal’s submissions come from African researchers,” he says. Likewise, *PLOS NTDs* offers an essential platform for researchers in LMICs—out of 4,702 papers published to date, 25% had at least one author from Africa, and 23% had an author from South America. Furthermore, 40% of the journal’s 255 editorial board members are from disease-endemic countries. Publishing work in open-access journals is not free, although *PLOS* and others waive or reduce fees for researchers from most LMICs, and some funders, such as the Bill & Melinda Gates Foundation, cover publishing costs for grantees. Nevertheless, the fees can prove problematic—although it is not a poor country, Morel notes that recent economic fluctuations have put Brazil’s scientists in a tough spot. “If I publish ten papers, my grant is exhausted,” he says. In this scenario, researchers may end up picking target journals based on price rather than impact.

Navigating peer review remains a daunting task, particularly for early-stage researchers or those for whom English is a second or third language. *PLOS NTDs* has conducted numerous writing workshops throughout the world to help researchers put together stronger manuscripts. “We talk about plagiarism, how to write the best article, why articles get rejected, and ethical issues in publications such as data management and data access,” says *PLOS NTDs* Co-Editor-in-Chief Serap Aksoy. “We’ve been very surprised by the need for—and lack of—information.” North–south collaborations can be advantageous in terms of breaking into the publishing world, as Makanga has observed for African researchers affiliated with the EDCTP. “We performed a bibliometric analysis, and the very collaborative researchers that we supported have very high impact publications—above the world average across all disease groups, and significantly more so in the area of HIV and TB,” he says [2].

## Making impact

Publishing is a key goal of TDR’s Structured Operational Research and Training Initiative, or SORT IT, program, which trains personnel from hospitals and government ministries to conduct robust, ethical, and actionable research with the public health data they have at hand. “If you have ten participants, then the minimum expectation is ten publications,” says Kamau. By 2015, the first iteration of SORT IT trained 269 individuals from 70 countries in the development of research projects related to diverse public health issues, including NTDs. Rose Jepchumba Kosgei of the University of Nairobi was one of the program’s earliest participants and found it highly rewarding in terms of getting her research into the literature. “I now have roughly 50 publications in peer-reviewed journals,” says Kosgei.

Many journals require that data be made available, but data sharing also has the potential to become a sore point for LMIC researchers. Gyapong points out concerns that well-funded scientists could profit unduly off the hard work of their African counterparts. “The trouble it takes to go out in the field and gather that kind of data is unimaginable,” he says. There is growing recognition of this issue, and although the International Committee of Medical Journal Editors has come out strongly in support of mandatory clinical research data sharing, it also noted that “the reasonable rights of investigators and trial sponsors must also be protected” and called upon the research community to develop equitable solutions for ensuring due credit and opportunities for collaboration [3].

Fortunately, African scientists are steadily gaining greater independence. “More and more African scientists are now leading studies, and instead of asking whether they’re getting credit, people are doing all the work,” says Malecela. African journals are also experiencing a resurgence, with many establishing an online presence and becoming indexed through services like PubMed. The African Journal Publishing Partnership is facilitating this process, fostering cooperation between African biomedical research titles and the editors of top-tier medical journals like *Lancet* and *JAMA*. Malecela has helped revitalize the *Tanzania Journal of Health Research* and notes that it has become a helpful outlet for early-stage researchers in her country. Although lacking the cachet and reach of their higher-profile international counterparts, these journals offer a home for research that Western reviewers may deem insufficiently “glamorous”—particularly in areas like implementation research, which focuses on things like how to design and deliver an effective public health intervention. “This is among the easiest research that can be conducted in LMICs,” says Kamau, “but some international journals feel this is ‘soft research.’”

This highlights a more fundamental conundrum about publication. Journal articles remain the core currency of science and a key indicator of “successful” capacity building, but science that appeals to reviewers and editors doesn’t necessarily produce direct public benefit. “You may publish something in *Nature* about discovering a gene, but implementation research, for example, is saving lives right now,” says Kamau. For this reason, SORT IT emphasizes translating research directly into policy briefs that are digestible by political leadership and thus more likely to have immediate effect. A well-crafted presentation can have a profound impact—for example, Malecela recalls presenting maps of lymphatic filariasis prevalence during an appearance of Tanzania’s Minister of Health before parliament in the early 2000s. “All of the members of parliament were saying, ‘My god, this is my district,’ and suddenly, it became a discussion of when the elimination program was going to start,” she says.

## Built to last

The most important element of scientific capacity building is ensuring that short-term investments produce long-term gains. Brain drain remains a serious issue, especially when LMICs train their best and brightest abroad. Saravia notes that Colombia’s scientific funding agency, Colciencias, spends roughly 60% of its budget on doctoral scholarships, many of which are used abroad, but does little to retain these students. “Colombia is an exporter of human talent—they don’t have programs for reinsertion,” she says, “and it’s not a return on public investment for the system if they don’t come back.” This is also a persistent problem in Africa, where many nations have an emigration rate of greater than 10% among highly educated citizens [4].

Accordingly, many training programs focus not just on an individual’s intellect but also on less tangible characteristics like their passion to help their country succeed. “It’s about the ‘chemistry’ of the individual and their personality and commitment,” says Molyneux. “You’ve got to have an individual who is prepared to put their career into this area.” There is also a growing focus on women, many of whom have missed opportunities to contribute because of cultural expectations that they will serve as the primary homemaker. In 2015, TDR issued a series of nine grants to female researchers across Africa to develop strategies for equalizing gender representation, including mentorship opportunities and workshops to build grant- and proposal-writing skills. Kamau points out that even simple solutions—like providing additional funding to pay for childcare—can have huge impacts.

Young scientists also need confidence that there are opportunities at home. Many LMIC institutions have sparse laboratory infrastructure and offer little funding outside of what researchers net from grants—in between heavy teaching and administrative obligations. “Our greatest limitations are a lack of funding and protected time from routine work for our research,” says Kosgei. Creating and sustaining these opportunities inevitably requires government involvement. Saravia notes that support from Colciencias was critical to the long-term survival of CIDEIM when international support declined, for example, and their backing helped facilitate its pursuit of outside funding. Nevertheless, it can be a struggle to persuade governments with limited resources to commit meaningful research funding—and as in any nation, political tides shift easily, and support can wax and wane with alarming regularity. But the simple act of investment has an important symbolic value, in Makanga’s view. “There should be a deliberate effort to be sure that countries coinvest, irrespective of the amount,” he says. “Coinvestment promotes local ownership, and it is key to the sustainability of the capacity that is being built.”

*Editor’s Note: David Molyneux, Carlos Morel, and Nancy Saravia serve as editorial advisors, and John Gyapong, Xiao-Nong Zhou, and Nilanthi de Silva serve as associate editors for *PLOS NTD*s. Mwele Malacela has published her work in this journal.
